# Self‐Reported Quality of Life and Lived Experiences of Adolescent Cancer Survivors Aged 10–19 in Southwestern Uganda: A Mixed‐Methods Study in a Resource‐Limited Setting

**DOI:** 10.1002/cnr2.70163

**Published:** 2025-03-04

**Authors:** Doreen Faith Longes, Leevan Tibaijuka, Moses Muwanguzi, Peter Kalubi, Steven Asiimwe, Kevin Schwartz, Howard Weinstein, Elizabeth Najjingo, Barnabas Atwiine

**Affiliations:** ^1^ Department of Paediatrics and Child Health Mbarara University of Science and Technology Mbarara City Uganda; ^2^ Department of Obstetrics and Gynaecology Mbarara University of Science and Technology Mbarara City Uganda; ^3^ Department of Psychiatry Mbarara University of Science and Technology Mbarara City Uganda; ^4^ Faculty of Medicine Mbarara University of Science and Technology Mbarara City Uganda; ^5^ Massachusetts General Hospital for Children Boston Massachusetts USA

**Keywords:** adolescents, cancer survivors, lived experiences, quality of life, Uganda

## Abstract

**Background:**

Cancer and its management affect the quality of life (QOL) and lived experiences of adolescent survivors.

**Aims:**

We describe the QOL and document the lived experiences of adolescent cancer survivors at a tertiary hospital in southwestern Uganda.

**Methods and Results:**

We conducted a mixed‐methods, cross‐sectional study using descriptive quantitative interviews using the summarized World Health Organization QOL questionnaire and qualitative in‐depth interviews with adolescent cancer survivors at Mbarara Regional Referral Hospital in southwestern Uganda in July and August 2023. We explored participants' perceptions of their health as a percentage of the overall QOL scores and evaluated their lived experiences using an inductive approach. The study obtained ethical approval from the Research and Ethics Committee of the Mbarara University of Science and Technology. A total of 42 adolescents with a mean age of 13.2 (SD ± 2.9) years participated in the study. Twenty‐three (55%) were males, and 24 (57%) had survived hematological malignancies. Participants reported very good (*n* = 12, 28.6%), good (*n* = 29, 69.1%), and poor (*n* = 1, 23%) QOL. Eleven (26.2%) and 30 (71.4%) participants reported they were very satisfied and satisfied with their health, respectively. Participants reported both positive and negative lived experiences. The positive experiences included persistent gratefulness, hope for a cure, and relationship restructuring. The negative experiences included concerns about body appearance, family separation, financial difficulties, and academic challenges.

**Conclusion:**

The QOL of adolescent cancer survivors in our setting is generally good and is influenced by support from family and the healthcare system. Their lived experiences are varied. Psychosocial services and peer support could improve perceived negative experiences.

**Abbreviations**
IDIs: in‐depth interviews
MRRH: Mbarara Regional Referral Hospital
MUST: Mbarara University of Science and Technology
PCU: Pediatric Cancer Unit
QOL: quality of life
WHOQOL‐BREF: World Health Organization Quality of Life Brief questionnaire

## 1 Background

Cancer diagnosis and its treatment can cause long‐term harm to the emotional, cognitive, and social development of adolescents [1, 2]. Many childhood cancer survivors experience chronic symptoms such as anxiety, fatigue, sleep disruption, pain, and cognitive deficits, which may negatively influence their quality of life (QOL) [3]. QOL is influenced by cancer type, age at diagnosis, duration of treatment, and gender [4].

Cancer diagnosis impacts the lives of most adolescent cancer survivors from the time of diagnosis, through the completion of therapy, and many struggle to cope with school and peers even after therapy [5]. In addition, others may struggle with cognitive impairment and psychosocial problems as a result of cancer [5]. Furthermore, the cognitive impairment caused by treatment side effects and procedures has been found to limit physical ability and emotional health and cause functional disorders [5]. In line with this, some of the negative effects include fear of cancer relapse, stigma and discrimination by peers or friends, fatigue, and depression, with some experiencing posttraumatic stress symptoms such as flashbacks and nightmares about the cancer [6]. They can also be distressed by their physical appearance from treatment‐related procedures and side effects such as skin changes, hair loss, surgical scars, and limb amputations [7]. However, some cancer survivors indicate that their diagnosis and treatment helped to create a positive view of life, good self‐esteem, improved self‐care and responsibility, and good interpersonal relationships, which are associated with posttraumatic growth [8].

The QOL and lived experiences of adolescent cancer survivors in low‐resource settings have not been adequately explored as most studies have focused on the evidence of disease remission or recurrence, with hardly any regard for psychosocial and environmental well‐being. The aim of the study was, therefore, to evaluate the QOL and document the lived experiences of adolescent cancer survivors who had completed cancer treatment for at least 3 months at the Pediatric Cancer Unit (PCU) of Mbarara Regional Referral Hospital (MRRH) in southwestern Uganda.

## 2 Methods

### 2.1 Study Design and Site

This was a cross‐sectional convergent mixed‐methods study involving both quantitative description of the QOL and phenomenological qualitative exploration of the lived experiences of adolescent cancer survivors at the PCU of MRRH. MRRH is a 600‐bed capacity specialized public facility located in Mbarara City, Southwestern Uganda, 260 km from the Capital, Kampala. The PCU is a 16‐bed capacity ward and outpatient clinic and the only pediatric cancer facility in southwestern Uganda, serving a population of over 10 million people and admitting an average of 120 children newly diagnosed with cancer per year. Cancer survivors are routinely seen in the clinic on appointments; their physical, social, and psychological status are evaluated during these visits, and appropriate interventions are made.

### 2.2 Study Participants and Eligibility Criteria

We included cancer survivors aged 10–19 years who had completed treatment for at least 3 months before the study and were registered at the PCU of MRRH. We included those who had spent 5 years after their cancer diagnosis and had completed their cancer treatment. We excluded any adolescent who had communication difficulties, making it hard for them to respond to questions.

### 2.3 Study Variables and Data Collection Tools

#### 2.3.1 World Health Organization Quality of Life—Brief Version (WHOQOL‐BREF)

This is a self‐administered tool consisting of 26 items, under four domains: physical health (7 items), psychological health (6 items), social relationships (3 items), and environmental health (8 items). The first two questions of the WHOQOL‐BREF were assessed separately: question 1, which asked about an individual's overall perception of QOL, and question 2, which asked about the overall satisfaction with their health. The domain scores are scaled in a positive direction on a 1–5‐Likert scale, where higher scores denote higher QOL. The mean score of items within each domain was used to calculate the domain score. The mean scores were then multiplied by 4 to make the domain scores comparable to the WHOQOL‐100 scores [9].

#### 2.3.2 Demographic Characteristics

These were assessed using the demographic questionnaire that collected information on age of adolescent (in years), sex (female, male), religion (Anglican, Catholic, Moslem, others), level of formal education (primary, secondary), tribe (Munyankore, Mukiga, Mukonjo, Others), primary caretaker (father, mother, others), type of family (extended, nuclear), and parents alive (both parents, father only, mother only).

#### 2.3.3 Clinical Characteristics

These factors included the cancer diagnosis, age at diagnosis (in years), duration on cancer treatment (in years), and history of relapse (yes, no).

For qualitative data, we used a semi‐structured interview guide to conduct in‐depth interviews (IDIs) with adolescents. The interview guide was developed with open‐ended questions and probes to explore the lived experiences among adolescent cancer survivors.

### 2.4 Study Data Collection Procedure

Eligible participants or their caregivers were contacted by phone and asked to participate in the study. Those who accepted were provided a date and time to come to the PCU. After written consent was obtained, interviews were conducted in a private room by a trained research assistant to collect participant sociodemographic characteristics. We allowed proxy reporting by caregivers where necessary.

After completing the quantitative questionnaire, IDIs were conducted with the adolescents who consented or assented to participate in these interviews. We reached saturation after 20 interviews, where no new lived experiences were reported by the adolescents during interviews. Participants for qualitative interviews were purposively sampled according to age, sex, cancer type, level of education, and duration of survivorship. All interviews were conducted in Runyankore/Rukiga and were audio‐recorded, and field notes were taken during the interview. The research assistants had prior experience in both quantitative and qualitative data collection procedures, with training in the responsible conduct of research. In addition, they were trained on key objectives of the study and the unique aspects relevant to adolescent cancer survivors.

#### 2.4.1 Data Management and Analysis

The quantitative data were entered into a Microsoft Excel 2010 spreadsheet and then exported to STATA 17 (StataCorp, College Station, Texas, USA) for data cleaning and analysis. The participant‐ and cancer‐related characteristics were summarized as frequencies and percentages (for categorical variables). Continuous variables (like age, age at diagnosis, duration on treatment) were presented as means and standard deviations (if normally distributed) and median and interquartile range (if not normally distributed). The Shapiro–Wilk test and histograms were used to assess for normality under the Gaussian assumption. The participants' overall perceptions of and satisfaction with health were also summarized as percentages of the overall QOL scores, and the different domain scores were reported as means, standard deviations, and/or medians and interquartile ranges.

All audio‐recorded qualitative interviews were transcribed verbatim and later translated into English, ensuring that information is not altered. English transcripts were critically reviewed by DFL and MM for accuracy of the information. DFL and MM read the transcripts in detail and carefully generated the initial codes that were reviewed by LT and BA for rigor. Any differences in data coding were resolved through discussion and consensus among the research team members. Data coding was done using the NVivo software package for qualitative data analysis [10]. Lived experiences of the participants were then analyzed using the inductive thematic approach.

#### 2.4.2 Ethics Statement

The study obtained ethical approval from the Research and Ethics Committee (REC) of the Mbarara University of Science and Technology (MUST) (MUST‐2023‐861). Written informed consent and assent were obtained from all the participants and their parents or guardians. Confidentiality, anonymity, and privacy were ensured throughout the study, and standard clinical practices and protocols were adhered to.

## 3 Results

A total of 66 eligible participants, only 42 adolescent cancer survivors whose caregivers were contacted, returned to the clinic and were asked to participate in the study, giving a response rate of 63.6%.

### 3.1 Participants' Demographic Characteristics

The mean age of the participants was 13.2 ± 2.9 years; 24 (57%) were early adolescents (10–13 years), 23 (55%) were male, and 35 (83%) were primary school pupils. Most participants had both parents still living (*n* = 33, 79%) and indicated mothers as their primary caretakers (64%).

### 3.2 Participants' Disease Characteristics

As shown in Tables 1 and 2, the mean age at cancer diagnosis was 9.9 ± 3.4 years; most of the participants had hematological malignancies (*n* = 24, 57%). Hodgkin lymphoma (*n* = 14, 33%) was the most common cancer type, followed by Wilms' tumor (*n* = 8, 19%). Half of the participants received only chemotherapy, while 26% received chemotherapy, surgery, and radiotherapy. Four participants (10%) had a history of cancer relapse.

**TABLE 1:** Demographic characteristics of adolescents.

Variable	Frequency (%)
Age (mean ± SD) in years	13.17 ± 2.89
Age categories (years)	
Early adolescent (10–13)	24 (57%)
Middle adolescent (14–17)	14 (33%)
Late adolescent (18–19)	4 (10%)
Sex	
Female	19 (45%)
Male	23 (55%)
Religion	
Anglican	16 (38%)
Catholic	18 (43%)
Moslem	3 (7%)
Others	5 (12%)
Level of formal education	
Primary	35 (83%)
Secondary	7 (17%)
Tribe	
Munyankore	19 (45%)
Mukiga	9 (21%)
Mukonjo	7 (17%)
Others	6 (14%)
Primary caretaker	
Father	6 (14%)
Mother	27 (64%)
Others	9 (21%)
Type of family	
Extended family	11 (26%)
Nuclear family	31 (74%)
Parents alive	
Both parents	33 (79%)
Father only	3 (7%)
Mother only	6 (14%)

**TABLE 2:** Disease characteristics of adolescent survivors.

Variable	Frequency (%)
Cancer diagnosis	
Hematological malignancies	24 (57%)
Hodgkin lymphoma	14 (58.3)
Non‐Hodgkin Lymphoma	7 (29.1)
Acute lymphoblastic leukemia	2 (8.3)
Acute promyelocytic leukemia	1 (4.1)
Nonhematological malignancies	18 (43%)
Wilms tumor	8 (44.4)
Germ cell tumor	3 (16.7)
Non‐rhabdoid soft tissue sarcoma	3 (16.7)
Osteosarcoma	2 (11.1)
Kaposi sarcoma	1 (5.5)
Ewing sarcoma	1 (5.5)
Mean age at diagnosis, years (SD)	9.9 (3.40)
Median duration of treatment, years (IQR)	1 (1–2)
History of relapse	
No	38 (90%)
Yes	4 (10%)

### 3.3 The Overall Perceptions and Satisfaction of Adolescent Cancer Survivors Regarding Their QOL and Health

**FIGURE 1:**
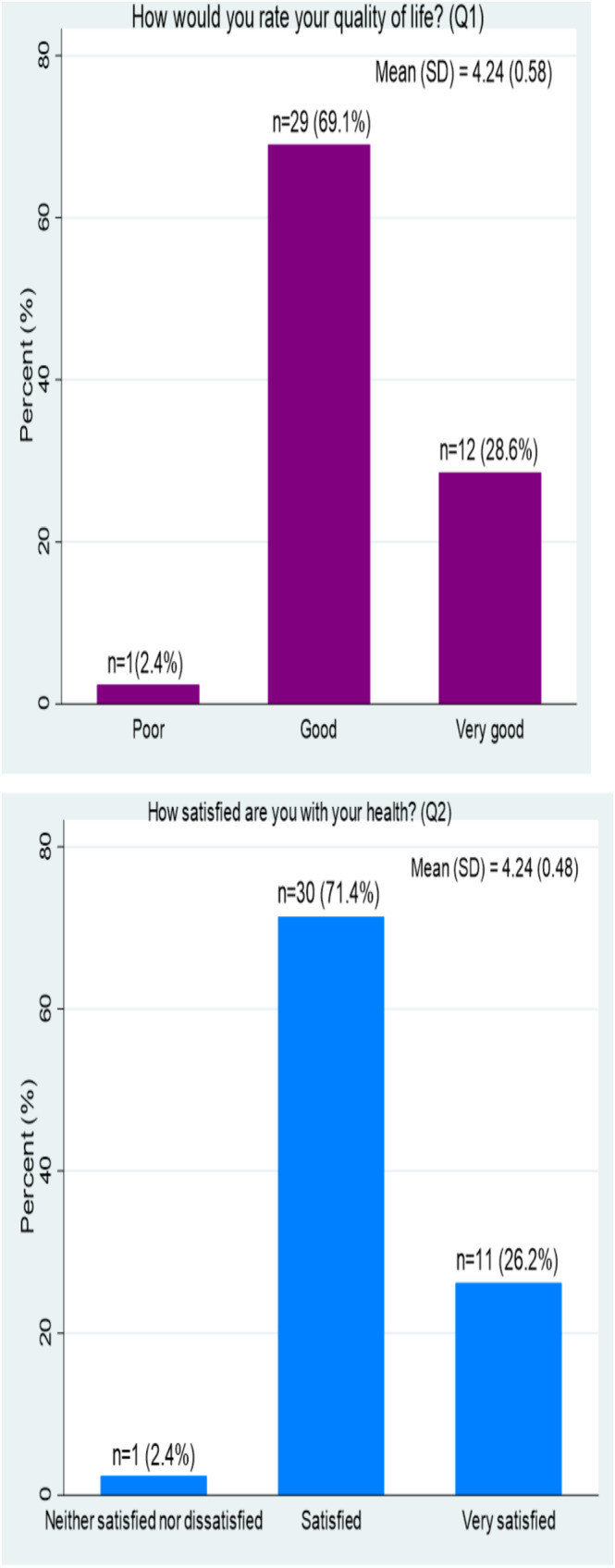
Overall perceptions and satisfaction of adolescent cancer survivors regarding their quality of life and health (questions 1 and 2 of the WHOQOL‐BREF scale), SD, standard deviation.

As shown in Figure 1, overall, only one participant rated their QOL as poor, while the majority reported it as good (69.1%) or very good (28.6%). Most of the survivors reported that they were either satisfied (71.4%) or very satisfied (26.2%) with their health (as assessed by questions 1 and 2 of the WHOQOL‐BREF questionnaire). The average scores were 4.2 (SD 0.58) and 4.24 (0.48) for questions 1 and 2, respectively.

### 3.4 Quality of Life Domains of Adolescent Cancer Survivors

**FIGURE 2:**
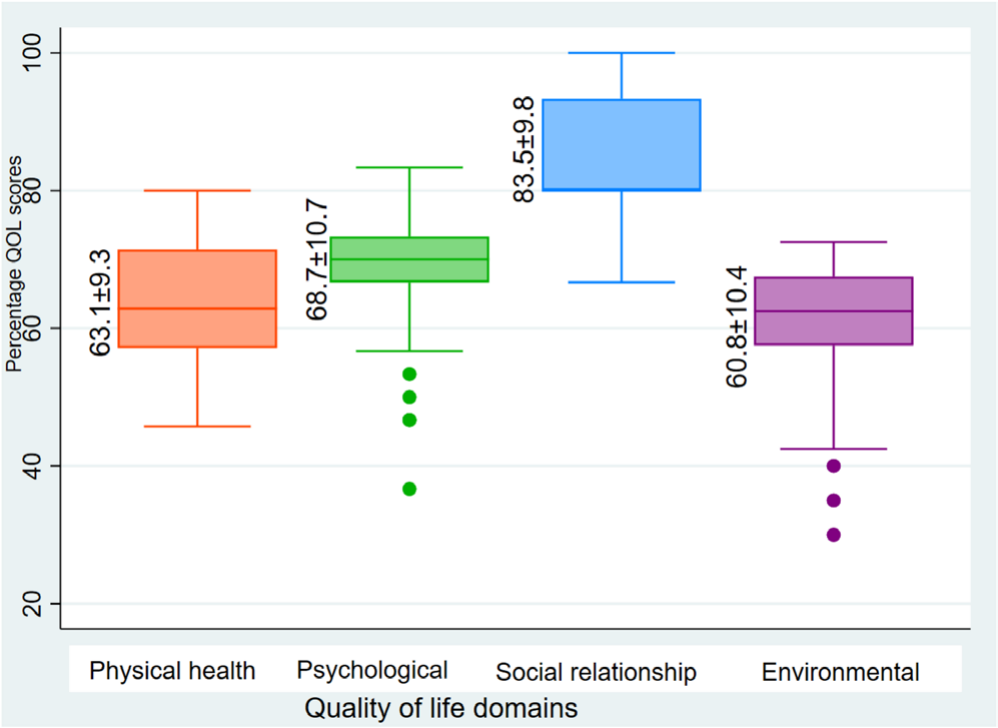
Box‐and‐whisker plot showing the QOL domain scores of the participants.

The mean scores (±SD) were as follows: physical health domain (57.1 ± 9.9), psychological domain (64.9 ± 11.2), social relationship domain (83.5 ± 9.8), and environmental domain (60.8 ± 10.4). The mean scores with SDs of the four domains of QOL are shown in Figure 2.

The assessment of the domain‐specific areas showed that physical health (*n* = 27, 64%) of the participants reported to be very much well able to get around with their life (*n* = 31, 74%) had a moderate amount of energy for everyday life (*n* = 25, 60%), were dissatisfied with their sleep, and (*n* = 32, 76%) were dissatisfied with their ability to perform their daily living activities. In the psychological health domain, the participants reported being extremely able to concentrate (*n* = 26, 62%), were dissatisfied with themselves (*n* = 25, 60%), and (*n* = 6, 14%) reported negative feelings such as blue mood, despair, anxiety, and depression very often. In the social relationship, health domain (*n* = 29, 69%) of the participants reported being satisfied with their personal relationships and relationships with the opposite sex (*n* = 25, 60%) together with the support they receive from friends (*n* = 26, 62%). In the environmental health domain, most of the participants (*n* = 27, 64%) reported feeling very much safe in their daily lives, had a moderate amount of money (*n* = 17, 41%), and were dissatisfied with access to health services (*n* = 28, 67%) and transport (*n* = 31, 74%).

## 4 Results of Thematic Analysis of the Qualitative Data

Two major themes emerged from the thematic analysis: negative and positive lived experiences with the respective subthemes as summarized in Table 3.

**TABLE 3:** Themes and subthemes from the thematic analysis.

Theme(s)	Negative lived experiences	Positive lived experiences
Subtheme(s)	Career change and lost need for career advancement	Persistent gratefulness
	Concerns of appearance and cosmesis	Living hope for cure of childhood cancer
	Family separation	Relationship restructuring
	Current expenditures and financial crisis	Power of religious leaders
	School and academic challenges	
	Fear and uncertainty of cancer relapse	

### 4.1 Theme: Negative Lived Experiences

In most interviews, most adolescent cancer survivors described experiencing many difficulties following the completion of cancer treatment. Most of these experiences affected their career advancement, family relations, perceptions of their bodily appearance, and continued need for medical follow‐up. Others reported experiencing anxiety and uncertainty, as indicated by the following subthemes.

### 4.2 Subtheme: Career Change and Lost Potential for Career Advancement

Many participants reported that they had to change their career aspirations to shorter courses such as vocational or hand‐skilled courses, which were not their initially desired career goals. Others were afraid of career limitations in terms of job acquisition due to the effects of cancer treatment, as reported by some of the survivors:

I had reached in primary five when I got sick. I had thought that I will go far at least secondary level four or six and join a course but I stopped in primary seven and joined a course, that's a challenge because my parents lost a lot of money in my treatment. I also felt hopeless and said to myself, nothing I will get up there, let me start from here (Participant 5).

### 4.3 Another Survivor Reported

I was fed up, my academics was a struggle because now I used to ask myself after school which job will I do now that I have only one limb but then the Doctor told me to focus on what I can manage and choose and focus on the goal I want to achieve, what my heart desires (Participant 7).

### 4.4 Subtheme: Concerns of Cosmesis and Appearance

Some participants described living in a state of unhappiness with their bodily appearance. Some female cancer survivors had concerns, especially with their skin, and worried about being unattractive.

I am just worried about my skin, it still has scars, and being a girl, my appearance concerns me. I need a tube that will remove those spots (Participant 16).

### 4.5 Subtheme: Family Separation

For some, cancer negatively affected some family relations, resulting in parental separation. Cancer was, in some cases, seen as a curse resulting in feelings of emotional trauma and unfulfilled needs

My dad left us with my mum saying he's tired of what was happening at home, cancer has never been in my family, so he got another woman and they rent in town. And there are some things that he used to give me, and do for me but now that stopped. Now my mother does everything, now she does even what she used not to do (Participant 14).

### 4.6 Subtheme: Current Expenditure and Financial Crisis

Some participants reported that their families lost property during their cancer treatment, which made their parents see no need for continuing medical follow‐up

At home … my grandmother used to rear goats but when I came here at the hospital, they kept selling them to provide money for support and now when they hear that I am coming to the hospital, they say … it is not relevant, you are fine, why don't you stop going back there, we don't have money for transport (Participant 17).

### 4.7 Subtheme: School and Academic Challenges

In most of the interviews, many participants reported a decline in their academic performance and a struggle to cope with their peers due to the effects of cancer:

I was performing well before, but now I am struggling and find it challenging because I used to miss school a lot. I feel bad that my friends have left me behind (Participant 4).

### 4.8 Subtheme: Fear and Uncertainty of Cancer Relapse

Some participants were anxious and worried about the possibility of cancer relapse, and this made them remain in a hopeless situation

(breathes deeply) My cancer coming back, that is what I fear most, and I don't want it (Participant 12).

### 4.9 Theme: Positive Lived Experiences

In most interviews, the participants expressed positive lived experiences following the completion of cancer treatment. Most of these experiences were related to posttraumatic growth and hope for a cure for their disease. These were mainly attributed to support from parents and relatives, healthcare workers, and religious leaders, as indicated by the following subthemes.

### 4.10 Subtheme: Persistent Gratefulness

Most participants were grateful for the continued support from their parents, relatives, and healthcare workers, which have given them the strength to overcome most of their fears

My mother and uncle did all they could to make me feel better and now I feel very good; they even still escort me to the hospital for follow‐up (Participant 9).

The nurses and doctors I found here were so nice to me and they used to treat me in time and talk to us nicely, which restored hope in my academics because I had lost interest at one point (Participant 6).

### 4.11 Subtheme: Living Hope for Curing Childhood Cancer

Some participants described feelings of giving hope to others who never believed cancer could be cured:

My friends used to say that cancer never gets cured but for me, I know am cured and they are now aware that cancer can get cured (Participant 20).

### 4.12 Subtheme: Relationship Restructuring

Most of the participants described how they had regained their lost relationships after surviving cancer:

When they knew I had cancer, many friends departed from me saying that I would spread to them (yet it's deadly) … Interestingly, the ones that had gone have started to come back because, they are seeing am doing everything very well (Participant 16).

### 4.13 Subtheme: The Power of Religious Beliefs

Some participants described living a life of compassion, especially for those with cancer, due to the influence of prayers they received from religious leaders when they had cancer and because their faith in God had also been enhanced

When I used to be here, priests used to come here and pray for me and pour anointing oil on my head. That truly gave me hope and I knew God would heal me. And now when I come for follow‐up and I see others with cancer, I pray for them in my heart and tell God to heal them too (Participant 19).

## 5 Discussion

The present study described the QOL of adolescent cancer survivors in addition to exploring their lived experiences. Almost all (97.7%) of our study participants reported either good (69.1%) or very good (28.6%) QOL, respectively, and the majority (97.6%) of the survivors were either satisfied (71.4%) or very satisfied (26.2%) with their health. These high QOL scores and health satisfaction are similar to what was found in Croatia in a study that reported an excellent QOL score of 90.1% among adolescents and young adults treated for cancer in childhood [11]. There is, however, a paucity of comparable data in low‐resource settings like ours.

High QOL scores and health satisfaction among adolescent cancer survivors in our study are both surprising and encouraging, considering the difficult social circumstances that characterize the lives of people in the developing world, especially those who have survived cancer. These adolescents have survived one of the worst health conditions and are grateful for their second chance at life. The frequent follow‐up appointments give them the rare opportunity to interface with the healthcare system that evaluates them and reassures them that their lives and health status are in good shape. As they described in the IDIs, these adolescents have had their family relationships restructured, are grateful for a second chance at life, and have developed good relationships with healthcare workers at the PCU, who offer them healthcare whenever they develop any symptoms, related or not related to the cancer they have survived. These participants also report receiving better attention from their caregivers and families and have good support from the healthcare system and friends, contributing to their perceptions of good QOL.

Additionally, our participants had high mean scores for psychological (64.9 ± 11.2), social relationship (83.5 ± 9.8), and environmental health (60.8 ± 10.4) domains, with lower scores for physical health (57.1 ± 9.9). These higher scores may be due to significant social support from families, especially parents, and peers, as some studies have reported that such support influences the QOL of children and adolescent cancer survivors [12]. Participants also had good social support and restored relationships with their friends, which could have also positively influenced their QOL, especially in the psychological, social relationship, and environmental health domains. A Norwegian population study also found higher psychological scores among adolescent cancer survivors [13]. However, in their study, the strengths and difficulties questionnaire, a psychosocial behavioral tool, was used, different from the WHOQOL‐BREF tool used in our study.

Conversely, our study found that adolescent cancer survivors had specific impairments in their physical health domain, and most reported having low energy for everyday life and were dissatisfied with their ability to perform daily activities and sleep. These findings are similar to those reported among adolescents and young adults in the united states (Pennsylvania) 1 year after their cancer treatment [14].

Participants in our study reported both negative and positive lived experiences as cancer survivors. Some reported having lost the ability to advance in their career or having changed their career aspirations due to the effects of cancer and its treatment. A study in Malaysia among cancer survivors aged 18–40 years revealed that cancer treatment significantly influenced career engagement and quality of work life among cancer survivors [15]. Although our participants were not a working population, their career aspirations were affected even after the completion of cancer treatment.

As much as financial crises are reported by survivors in most studies, the fears are different. Our participants were more worried about the money and property lost during cancer treatment by their families and reported the urge to compensate for the costs incurred during their treatment. In other studies, however, cancer survivors reported job‐related stress: fear of being fired, health insurance coverage, and related financial concerns as their major worry [16]. That study, however, looked at young adults aged 18 years and above, whereas our study looked at adolescents aged 10–19 years, a population that was mostly not yet working in most cases.

Like in other studies [17], our study showed that the stress of families going through the treatment of their child's cancer can lead to divorce and/or separation and other hardships in relationships, worsening an already difficult situation for cancer survivors. This can further affect their coping mechanisms and school performance.

There were some positive lived experiences reported in our study, and subthemes such as persistent gratefulness, living hope for the treatment of childhood cancer, relationship restructuring, and the power of religious leaders were identified.

The majority of our study participants reported a lived life of gratefulness for the social support they received from their parents, relatives, and healthcare workers before and after cancer treatment, which has given them the hope and strength to overcome challenges. Many studies have emphasized the impact of social support on improving the QOL of adolescent cancer survivors [16]. Families, especially parents and friends, are a great source of emotional, motivational, and practical social support that influences the lives of adolescent cancer survivors [18]. Some participants reported a restoration of their relationships with their friends after the completion of their treatment. Support from healthy peers is very important for enhancing psychosocial support and school reentry among adolescent cancer survivors [19], which, in turn, makes them compassionate toward other children with cancer. Additionally, religious beliefs and spirituality are a means of adaptation and coping and sources of resilience for cancer survivors [20].

## 6 Strengths and Limitations

This study has certain limitations. First, this study was carried out at a single PCU, which may not be a representative of cancer survivors in settings different from ours. Second, younger adolescents were unable to share constructive experiences, and most of the study's substantive information was obtained from older adolescents. Thirdly, responses to the QOL questionnaire might be affected by social desirability bias resulting in higher mean QoL scores. However, in our cultural context, this could point to resilience developed by these adolescents following cancer survivorship as described in their positive lived experiences. Fourth, we did not explore all factors that affect the QOL of participants, like the educational or peer support structures. Despite these limitations, using a mixed‐methods approach helped us to understand some of the major issues experienced by adolescent cancer survivors that impact their QOL.

## 7 Conclusion

The QOL reported by adolescent cancer survivors in our settings is generally good as was their satisfaction with their overall health. A variety of lived experiences, both negative and positive, were described by the adolescent cancer survivors in this study. Psychosocial services and ongoing survivorship support could provide support to mitigate negative experiences in this population. In addition, key stakeholders like the professional bodies of hemato‐oncologists, hospice, and ministry of health may need to design and implement policies focusing on improving survivorship among children with cancer. This may include supporting the treatment process and provision of psychosocial support to the parents and their families to fully activate the adolescent support systems during and after cancer treatment.

## Author Contributions

**Doreen Faith Longes:** conceptualization (lead), data curation (lead), formal analysis (lead), funding acquisition (lead), investigation (lead), methodology (lead), project administration (lead), software (supporting), supervision (lead), validation (lead), visualization (lead), writing – original draft (lead), writing – review and editing (lead). **Leevan Tibaijuka:** data curation (supporting), formal analysis (supporting), methodology (supporting), resources (supporting), software (lead), writing – review and editing (supporting). **Moses Muwanguzi:** data curation (supporting), formal analysis (supporting), methodology (supporting), software (supporting). **Peter Kalubi:** supervision (supporting). **Steven Asiimwe:** validation (supporting), visualization (supporting), writing – review and editing (supporting). **Kevin Schwartz:** validation (supporting), visualization (supporting), writing – review and editing (supporting). **Howard Weinstein:** validation (supporting), visualization (supporting), writing – review and editing (supporting). **Elizabeth Najjingo:** methodology (supporting), project administration (supporting), supervision (supporting), validation (supporting), visualization (supporting), writing – review and editing (supporting). **Barnabas Atwiine:** conceptualization (supporting), funding acquisition (supporting), methodology (supporting), project administration (supporting), resources (supporting), supervision (lead), validation (lead), visualization (lead), writing – original draft (supporting), writing – review and editing (supporting).

## Conflicts of Interest

The authors declare no conflicts of interest.

## Data Availability

The data that support the findings of this study are available from the corresponding author upon reasonable request.

## References

[1] C. Eiser , “Beyond Survival: Quality of Life and Follow‐Up After Childhood Cancer,” Journal of Pediatric Psychology 32 (2007): 1140–1150. 17644535 10.1093/jpepsy/jsm052

[2] A. Rueda , J. Macintyre , and A. Thomassen , “Challenges Faced by Adolescent and Young Adult Patients With Cancer,” Oncology Nursing News 16 (2022): 31–32.

[3] L. L. Robison and M. M. Hudson , “Survivors of Childhood and Adolescent Cancer: Life‐Long Risks and Responsibilities,” Nature Reviews Cancer 14 (2014): 61–70. 24304873 10.1038/nrc3634 PMC6425479

[4] U. M. Sansom‐Daly and C. E. Wakefield , “Distress and Adjustment Among Adolescents and Young Adults With Cancer: An Empirical and Conceptual Review,” Translational Pediatrics 2 (2013): 167. 26835313 10.3978/j.issn.2224-4336.2013.10.06 PMC4729076

[5] K. Kim and H. Yoon , “Health‐Related Quality of Life Among Cancer Survivors Depending on the Occupational Status,” International Journal of Environmental Research and Public Health 18 (2021): 3803. 33917318 10.3390/ijerph18073803 PMC8038705

[6] C. Wayant , J. Manquen , H. Wendelbo , et al., “Identification of Evidence for Key Positive Psychological Constructs in Pediatric and Adolescent/Young Adult Patients With Cancer: A Scoping Review,” Journal of Adolescent and Young Adult Oncology 10 (2021): 247–259. 33464990 10.1089/jayao.2020.0184 PMC8220547

[7] G. P. Quinn , V. Gonçalves , I. Sehovic , M. L. Bowman , and D. R. Reed , “Quality of Life in Adolescent and Young Adult Cancer Patients: A Systematic Review of the Literature,” Patient Related Outcome Measures 6 (2015): 19. 25733941 10.2147/PROM.S51658 PMC4337625

[8] G. Engvall , M. Cernvall , G. Larsson , L. von Essen , and E. Mattsson , “Cancer During Adolescence: Negative and Positive Consequences Reported Three and Four Years After Diagnosis,” PLoS One 6 (2011): e29001. 22194973 10.1371/journal.pone.0029001 PMC3237575

[9] S. M. Skevington and C. Tucker , “Designing Response Scales for Cross‐Cultural Use in Health Care: Data From the Development of the UK WHOQOL,” British Journal of Medical Psychology 72 (1999): 51–61. 10194572 10.1348/000711299159817

[10] QSR International , “QSR International. NVivo [computer software],” Version 14. 2023, https://www.qsrinternational.com/nvivo‐qualitative‐data‐analysis‐software/home.

[11] M. Sedmak , A. Bogdanić , and M. Grubić , “Correlates of Quality of Life in Pediatric Cancer Survivors,” Psychiatria Danubina 32 (2020): 533–539. 33212460

[12] S. A. Schepers , K. Russell , K. S. Berlin , H. Zhang , F. Eang , and S. Phipps , “Daily Mood Profiles and Psychosocial Adjustment in Youth With Newly Diagnosed Cancer and Healthy Peers,” Health Psychol 39, no. 1 (2020): 1–9. 31682149 10.1037/hea0000810

[13] A. C. Mertens and J. G. Marchak , “Mental Health Status of Adolescent Cancer Survivors,” Clinical Oncology in Adolescents and Young Adults (2015): 87–95.

[14] L. C. Daniel , R. Aggarwal , and L. A. Schwartz , “Sleep in Adolescents and Young Adults in the Year After Cancer Treatment,” Journal of Adolescent and Young Adult Oncology 6 (2017): 560–567. 28628351 10.1089/jayao.2017.0006

[15] S. R. A. Hamzah , S. N. S. Musa , Z. Muda , and M. Ismail , “Quality of Working Life and Career Engagement of Cancer Survivors: The Mediating Role of Effect of Disease and Treatment,” European Journal of Training and Development 45 (2021): 181–199.

[16] S. L. Crowder , R. Sauls , L. M. Gudenkauf , et al., “The Lived Experience of Young Adult Cancer Survivors After Treatment: A Qualitative Study,” Nutrients 15 (2023): 3145. 37513563 10.3390/nu15143145 PMC10385438

[17] L. Mader , M. Hargreave , L. E. Frederiksen , et al., “The Impact of Childhood Cancer on Parental Separation, Divorce, and Family Planning in Denmark,” Cancer 126 (2020): 3330–3340. 32449155 10.1002/cncr.32901

[18] M. H. Larsen , E. H. Larsen , E. Ruud , A. Mellblom , S. Helland , and H. C. Lie , “‘I Have to Do Things Differently Now, but I Make It Work’—Young Childhood Cancer Survivors' Experiences of Self‐Management in Everyday Living,” Journal of Cancer Survivorship 16 (2022): 728–740. 34097249 10.1007/s11764-021-01066-y PMC9300523

[19] M. V. Ingersgaard , M. K. Fridh , T. Thorsteinsson , L. Adamsen , K. Schmiegelow , and H. Baekgaard Larsen , “A Qualitative Study of Adolescent Cancer Survivors Perspectives on Social Support From Healthy Peers–A RESPECT Study,” Journal of Advanced Nursing 77 (2021): 1911–1920. 33470450 10.1111/jan.14732

[20] C. Chen , X. Sun , Z. Liu , M. Jiao , W. Wei , and Y. Hu , “The Relationship Between Resilience and Quality of Life in Advanced Cancer Survivors: Multiple Mediating Effects of Social Support and Spirituality,” Frontiers in Public Health 11 (2023): 5–6. 10.3389/fpubh.2023.1207097 PMC10493315 37701908

